# A Trustable Spine Abnormalities Classification System Using ResNet50 and VGG16 Supported by Explainable Artificial Intelligence

**DOI:** 10.3390/biomimetics11030206

**Published:** 2026-03-12

**Authors:** Muhammad Shahrul Zaim Ahmad, Nor Azlina Ab. Aziz, Heng Siong Lim, Anith Khairunnisa Ghazali, Mubashir Ahmad, Farshid Amirabdollahian, Afif Abdul Latiff, Kamarulzaman Ab. Aziz

**Affiliations:** 1 Faculty of Engineering & Technology, Multimedia University, Melaka 75450, Malaysia; 1181101174@student.mmu.edu.my (M.S.Z.A.); hslim@mmu.edu.my (H.S.L.); 2 Centre for Advanced Analytics, COE Artificial Intelligence, Multimedia University, Melaka 75450, Malaysia; anith.ghazali@mmu.edu.my; 3 Robotics Research Group, University of Hertfordshire, Hatfield AL10 9AB, UK; m.ahmad21@herts.ac.uk (M.A.); f.amirabdollahian2@herts.ac.uk (F.A.); 4 Faculty of Medicine, University Kebangsaan Malaysia, Kuala Lumpur 56000, Malaysia; afif@hctm.ukm.edu.my; 5 Faculty of Business, Multimedia University, Melaka 75450, Malaysia; kamarulzaman.aziz@mmu.edu.my

**Keywords:** explainable artificial intelligence, heatmaps, spine, spondylolisthesis and scoliosis

## Abstract

Deep learning has been applied in various fields and has been proven to provide good results for classification tasks. However, there is limited understanding of a deep learning model’s decisions, so deep learning is commonly described as a black box. Applying deep learning for critical applications such as medical diagnostic process introduces trust issues. For the deep learning model to be trusted by the medical practitioners, the methods employed by the deep learning model must be seen to be aligned with the diagnostic process employed by the medical practitioners. Explainable methods such as Grad-CAM can be applied to improve the explainability of the deep learning models by providing an visual interpretation of the deep learning classification result decision process. In this study, two deep learning models, VGG16 and ResNet50 are trained using three training methods, one with randomly initialized weights, and two transfer learning methods, which are feature extraction and fine-tuning, to classify the spinal abnormalities based on X-ray images. The classification metrics results are compared and further analyses using Grad-CAM heatmaps are included. The models also evaluated using a stratified five-fold cross-validation, results revealed some disparity between the model’s accuracy and clinical relevance. The randomly initialized VGG16 obtained a classification accuracy of 93.79% but does not focus on clinically relevant regions. On the other hand, not only do the fine-tuned ResNet50 and VGG16 obtain high accuracies of 98.22% and 99.12%, but the heatmaps show that the models focus on more relevant regions. A comparison of the two models shows that the heatmaps produced by the fine-tuned ResNet50 are in more agreement with the clinical view than the fine-tuned VGG16. This study provides a useful reference for interpreting a deep learning-based classification result using explainable method particularly in spine abnormalities analysis with Grad-CAM.

## 1. Introduction

The origin of deep learning models, artificial neural networks (ANNs), is inspired based on how the biological brain learns [1]. Computer scientists designed ANN algorithms to mimic the brain’s ability to learn by recognizing patterns and experiences. Deep learning is a subfield of machine learning that focuses on deep neural networks, where it utilizes hierarchical representations to learn from data, where the network layers can range from a few to hundreds [2]. However, deep learning is not limited to neuroscientific-inspired models. Examples of deep learning models are convolutional neural networks (CNNs), Recurrent Neural Networks (RNNs) [3], and transformers [4]. Deep learning has been successfully applied in various fields such as facial recognition, weather forecasting, financial planning, robotics, precision agriculture planning, and medical image analysis. The successful design and implementation of deep learning models is highly reliant on the advancement in computing software and hardware.

However, the only visible outcome of a deep learning model is the output, which is generated based on the learned features stored in the network weights. Due to these issues, deep learning models are commonly referred to as black boxes, where the internal mechanisms are not interpretable by humans [5]. Thus, this posed an issue for critical applications, such as in medical tasks where interpretability is crucial. With this issue, an Explainable Artificial Intelligence (XAI) program was started by the Defense Advanced Research Projects Agency (DARPA) to allow users to effectively use, manage, understand, and subsequently trust artificial intelligence (AI) models [6]. The program concluded that currently, there are no universal methods for XAI. It was observed that different users need different explanations for the predictions made by AI models.

The explainability of AI is extremely important in the development of AI-enabled medical systems. For the deep learning predictions to be trusted by the medical professionals, the result must align with the diagnostic methods employed by the doctors. The model developed should be able to analyze the data in the same way a medical expert analyzes the data. In medical image analysis, the model must look at the same key features as those looked at by the doctors. The trustworthiness issue of a medical AI system may occur when a model is trained for a classification task using limited images. Even though acceptable accuracy is achieved, it is possible that the model achieved this without exactly learning the correct features required to diagnose the condition of the subject in the image. Additionally, there is also the issue of overfitting, where the model is adapted specifically to the data used for training and testing the model, but performs worse on unseen data. Due to this issue, a visualization of the decision process would assist in deciphering what the deep learning models see.

In image analysis, a heatmap highlighting regions that contribute to the classification result. It can be used as an explanation to support the result [7]. Class activation mapping (CAM) was proposed by Zhou et al. [8] to identify the region that contributes to the class-specific prediction made by the deep learning model for the classification task. Different CAM-based methods were developed in the past years, such as Gradient-weighted CAM (Grad-CAM) [9], Score-CAM [10], Recipro-CAM [11], ICR-CAM [12] and Grad-CAM++ [13]. The CAM methods enable users to reason with the predictions made, thus increasing the explainability.

This work focuses on the explainability of AI models developed to classify spine abnormalities, specifically, spondylolisthesis and scoliosis. Spondylolisthesis is a condition where the vertebrae slide out of the proper position, while scoliosis is a sideways curve of one’s spine, which might be caused by spondylolisthesis. In this study, transfer learning methods, which include feature extraction and fine-tuning, are used to classify the X-ray images of the spine as either normal, spondylolisthesis, or scoliosis.

Transfer learning is the application of a model trained using different sets of data, which are usually larger in size. Transfer learning is reportedly to give a better performance compared to training a new model from scratch [14]. Many pretrained image classification models are made available from the ImageNet Large Scale Visual Recognition Challenge (ILSVRC). The pretrained deep learning models tested in this study are Residual Network (ResNet) and Visual Geometry Group (VGG). The models are originally trained using ImageNet dataset with more than 1 million images with a vast number of classes. Both ResNet and VGG are used here to classify X-ray images from an open dataset into the three classes. After training the models using transfer learning, the Grad-CAM heatmaps are overlaid onto the original images to reveal the areas that contribute most to class prediction. The heatmaps are analyzed by an orthopedic surgeon on the team to verify the relevance of the features highlighted by the models. Both pretrained models are evaluated as-is and after fine-tuning on the dataset. The Grad-CAM heatmaps show that fine-tuning improves the models’ focus on relevant image regions. Additionally, although the accuracy of ResNet is found to be higher than that of VGG, the Grad-CAM heatmaps show that VGG examines areas more closely aligned with how medical practitioners analyze these images.

The main objectives of this study are:

- To develop and benchmark ResNet-50 and VGG-16 for multiclass classification of spinal X-ray into normal, scoliosis, and spondylolisthesis.
- To evaluate the impact of transfer learning by comparing the performance of models trained from scratch versus transfer learning models fine-tuned on the specific spinal dataset.
- To implement XAI (Grad-CAM) to visualize and interpret the model’s decision of the black-box models and ultimately identify a trustworthy model.

The contributions of this study are:

1. Application of ResNet and VGG transfer learning models for the spine abnormalities classification, specifically spondylolisthesis and scoliosis, from plain X-ray images.
2. Implementation of Grad-CAM to visualize and interpret the results from deep learning models in a medical context.
3. A comparative study demonstrates that higher classification accuracy (VGG16) does not necessarily correlate with better visual interpretability (ResNet50).
4. Verification of the Grad-CAM heatmaps generated by a medical expert to establish clinical relevance.

The rest of this paper discusses the study further. Specifically, following Section 1, related works are reviewed in Section 2. Then, the methodology applied in this work is presented in Section 3, followed by the results and their discussion in Section 4. The work is concluded in Section 5.

## 2. Related Works

Despite the many proposed works on AI usage for the medical sector, the adoption rate does not match the development progress. The trust issue among the various stakeholders is the reason for slow adoption [15]. Among the factors affecting trust are transparency and explainability of the system. Explainability provides meaningful information to medical experts on how AI makes a decision. The ability to understand the reasons behind the AI decision improves trust in AI usage in the medical field [16].

Heatmaps are usually used in XAI, especially for image data. They provide visualizations that offer reasoning and may help increase trust in AI-based medical diagnosis. In medical applications, heatmaps are commonly used to highlight important areas for detecting the locations of pathologies. Kim et al. [17] demonstrated that heatmap visualization using Grad-CAM can be used for hemorrhage detection and localization. The authors found that most of the heatmaps were able to locate the location of hemorrhage in the correct predictions. The authors measure the displacement distance between the highest value of the Grad-CAM heatmap and the center pixel hemorrhage. The model utilized in the hemorrhage detection was ResNet50. Three deep learning architectures, VGG, ResNet, and ChexNet, are compared by Vardhan et al. for lung opacities estimation from chest X-rays [18]. The models were pretrained, and two-stage training processes were implemented. The first stage only trains the classifier while the base convolutional layers are frozen. The second stage unfreezes a few layers in the base layers to be trained together with the fully connected layers. The heatmaps are generated after training the models. A new method, the heatmap concordance score, was introduced to calculate the area of overlap between the heatmap and the lungs.

Generally, heatmaps are commonly used to visualize the features that the models seem to focus on most when making predictions of a certain class. The heatmaps are used to visualize the important features within the images that are important for classification decisions of magnetic resonance imaging (MRI) images in the study by Gai [19]. The classification task consists of 10 classes and is done using a 71-layer model based on ResNet18. The study found that the focused regions shown by the heatmaps correspond well with the expert’s perception. Heatmaps are used to visualize the hill classification of the gastroesophageal valve using ConvNet [20]. Heatmaps are also seen for inspection of colon classification tasks, such as colon cancer [21] and colon conditions [22]. In the work by Li et al. [23], Grad-CAM is applied, and the features extracted for Alzheimer’s disease diagnosis are found to be relevant, as verified by a medical expert.

Beyond soft tissue analysis, XAI has increasingly been applied to musculoskeletal tasks. The musculoskeletal classification task using VGG16 is used for osteoporosis detection by Jang et al. [24]. Additionally, Grad-CAM is used to visualize the prediction. Sarmadi et al. compared different deep learning architectures with the Vision Transformer (ViT) [25,26]. The deep learning models utilized in the paper are ResNet and VGG. The heatmaps are compared to observe the reliability of the deep learning models in identifying the areas affected by osteoporosis. The authors found that ViT can locate the affected areas more effectively in comparison to CNN. Additionally, Grad-CAM heatmaps are also used to validate the classification result, highlighting the areas in the images where the deep learning is focused on. It was observed that the region highlighted from Grad-CAM heatmaps matches the relevant regions reviewed by orthopedic specialists [27,28]. Furthermore, VGG16, ResNet50, and InceptionV3 are used in lung cancer classification in [29]. The models’ performance in locating high-level features that influence the decision is analyzed with Grad-CAM.

In the specific domain of spinal analysis, recent studies have achieved high classification accuracy, yet interpretability remains a challenge. For example, Güneş et al. [30], and Saravagi et al. [31] reported high accuracies (ranging from 92% to 99%) for scoliosis and spondylolisthesis classification using models like VGG, ResNet, InceptionV3, and EfficientNet. However, neither study implemented XAI methods to validate these high metrics. On the other hand, Polis et al. utilized a Vision-Language Model (VLM), specifically BiomedCLIP, for classification [32]. Unlike traditional training methods, the authors employed a zero-shot approach, utilizing the model’s pretrained Vision Transformer (ViT) and biomedical text to detect scoliosis and the severity without task-specific fine-tuning. Nevertheless, no XAI method is implemented to validate the visual features. A paper by Chen et al. discussed the application of Grad-CAM for interpreting the spondylolisthesis diagnosis result using EfficientNetV2 [33]. The models obtained 92.0% accuracy. The authors noted that visualization can be used to interpret the classification prediction and would assist in clinical settings.

When XAI is applied to spine analysis, results have been mixed. ResNet-50 is used for semi-supervised classification of lumbar spine herniation in the work done by Hou et al. [27]. Grad-CAM heatmaps of the images are then generated to analyze the rationality of the results produced by the model. The Grad-CAM managed to identify important regions that are important for the predicted class. However, further qualitative analysis is not present. Similarly, He et al. analyzed the heatmap generated from the deep learning models and found that the heatmaps focus on the apical vertebra, which is an important feature of scoliosis [28]. Additionally, Takahashi et al. utilized a variety of deep learning models as an ensemble to determine the classification result for curve progression in adolescent idiopathic scoliosis, which revealed inconsistent Grad-CAM activation across different models [34]. The models include ResNet50, DenseNet121, InceptionV3, ConvNextV2, ViT, and SwinT. The heatmap displays predictions from CNN models that identify spine anatomical features better than transformer models. However, all models present inconsistent activation maps, with the heatmaps not only focusing on the spinal columns, but also on irrelevant features such as background and other organs. An article by Lu et al. discusses the performance of Xception in the diagnosis of scoliosis and spondylolisthesis [35], where the partition explainer is used by the authors as an XAI method. However, further analysis of the partition explainer was absent.

From the works reviewed, although heatmaps, particularly Grad-CAM, are commonly used to verify and explain the model’s decision-making process, there are no universal metrics to qualitatively or quantitatively evaluate a model trustability using heatmaps generated. However, expert opinions are often valued to verify the heatmaps’ classification features when making decisions. Based on the review of related works, while deep learning has shown promise in spinal abnormalities classification, several gaps remain. The motivations for this study are summarized as follows:

1. Inconsistency in XAI studies: While some studies claim that deep learning models focus on spinal regions, some display that heatmaps focus on irrelevant regions (background noise, organs), highlighting a need for more robust training strategies to ensure clinical relevance and enable direct comparison between deep learning models.
2. Absence of comparative training strategy analysis: There is a lack of research specifically comparing how different training methods, specifically random initialization (training from scratch) and transfer learning, can impact the explainability of the model in the spinal domain. Most comparisons focus solely on quantitative performance, neglecting the impact of training methods on visual attention maps.

To address these gaps, this research benchmarks ResNet50 and VGG16 using Grad-CAM. Additionally, we evaluate how the choice of training strategy influences not just the accuracy, but the interpretability of the model, with validation by a medical expert to bridge the gap between computational metrics and clinical trust.

## 3. Methodology

### 3.1. Deep Learning Models Training and Evaluation

The dataset utilized in this study is X-ray images of vertebrae collected by the King Abdullah University Hospital, Jordan University of Science and Technology. The data is made available on Kaggle [36]. The dataset consists of 338 subjects (98 males, 240 females). In the dataset, three classes are present, namely normal, spondylolisthesis, and scoliosis. The number of images for normal spines is 71, while the number of spine images diagnosed with spondylolisthesis is 79, and the number of spine images diagnosed with scoliosis is 188 (see Table 1).

The training method implemented in the study is a stratified 5-fold cross-validation (CV). Each of the models is trained five times on each fold using different training–validation splits. Each fold uses 80% of the images for training (training set) and evaluation on the remaining 20% images (validation set). As a result, all images are used for evaluation exactly once. The final performance metrics were calculated by aggregating the validation predictions across five folds.

The resolution and aspect ratios of the images in the dataset are not uniform. Therefore, all the images are resized to a similar size of 224 × 224 with RGB color channels. Multiple image transformation methods are applied to the training set to mitigate overfitting. The transformations are applied to artificially increase the data size, commonly known as data augmentation. The geometric transformation methods include the random horizontal flip function, where the images are mirrored with respect to the vertical axis, and a random rotation function that rotates the images within 15 degrees. Random affine transformation is applied with a translation value of ±10% for both the x-axis and y-axis. This enables the models to locate important regions at different locations, increasing the model’s ability to generalize. Additionally, a scaling factor of between 80% and 120% with a shear value of −10° and +10° is specified for the random affine transformation function. The scaling factor and shear functions are introduced to teach the models to recognize important regions of different sizes and to increase the model’s robustness to new data. For random affine transformation, the values indicate the range for random transformations that are applied for each epoch cycle to each image.

In addition, photometric transforms are also applied to reduce the model dependency on predicting the classes according to the color information. A photometric transformation using random color jitter is applied to the model. Then, the images are normalized by converting to a tensor by rescaling from [0, 255] to [0.0, 1.0] to standardize the input scale. Finally, mean–standard deviation normalization is applied to the images according to the mean and standard deviation values of the ImageNet dataset. The transformation methods implemented in this study are from the TorchVision library (v0.19.0).

The models trained and evaluated in this study are ResNet-50 and VGG16. ResNet-50 is a variation of the Residual Network (ResNet) that includes 50 layers [37]. ResNet employs shortcut connections, which is called deep residual learning. In this study, ResNet-50 is chosen as a middle ground between deeper ResNet models, such as ResNet-152, and shallower models, which is ResNet-18. ResNet-50 provides a balance between performance and computational cost. Next, VGG16 is a variation of a deep convolutional network released by the Visual Geometry Group in 2014 [38]. The model was considered very deep when it was released and performed quite well during the ImageNet Challenge in 2014 [39]. The VGG model consists of 5 convolutional blocks, and each block is followed by a maxpooling layer. VGG16 consists of 13 convolutional layers and 3 fully convolutional layers, making the total number of layers with trainable weights equal to 16. Both models used were previously trained using the ImageNet dataset, which includes 1000 classes of objects and animals with more than one million images [40]. The models are loaded from the PyTorch library.

**Figure 1:**
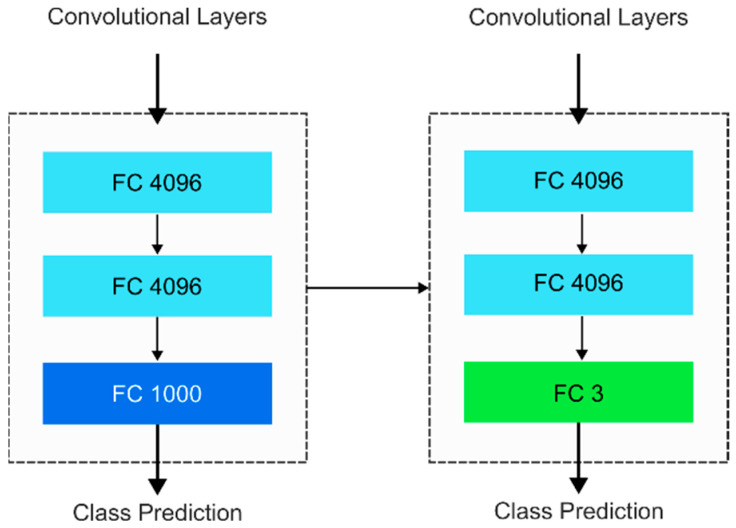
VGG-16’s original classifier (**left**) is replaced with a new classifier (**right**). The original final layer (dark blue) for 1000-class classification is replaced with a new classification layer for three classes (green).

Three training methods utilizing transfer learning are applied in this study. The first method utilized randomly initialized weights of the models, and the remaining two are variations of transfer learning, which are feature extraction and fine-tuning of the pretrained models. Table 2 summarizes the differences between the training strategies. The original classifier in both transfer learning models was trained to classify 1000 classes and was replaced with a new classifier for three classes, which are spondylolisthesis, scoliosis, and normal spine. The original classifier and the new classifier are displayed in the image displayed in Figure 1. The new classifier for ResNet50 and VGG16 is similar to the original classifier, except that the last layer, a dense layer consisting of 1000 neurons for the ImageNet classification task, is replaced with a dense layer consisting of three neurons to accommodate a three-class classification task.

For the feature extraction using the transfer learning method, the base layers of the models are frozen. All the weights in the convolutional layers of the models are identical to those of the model trained for the ImageNet classification task. Only the classifiers are trained. This enables knowledge transfer from the pretrained networks to detect similar features in the new dataset. This is especially useful for a new dataset that is considered similar to the dataset the models were previously trained on. Additionally, it is also reported by Chu et al. that, when the data is small, and the new data is similar to the trained data, the feature extraction method outperforms the fine-tuning method [41].

For the classification tasks using data that are not related to the original dataset of ImageNet, such as pathological classification from spine X-ray images, the model might not be able to focus on relevant features in making decisions [42]. To solve this, fine-tuning of the pretrained model can be applied [2,43]. For the fine-tuned model, the initial weights for each of the models are based on the model trained on the ImageNet dataset. However, during the training process of the fine-tuned model, the weights of the classifiers and base layers are unfrozen and updated using our dataset. The updated weights in the convolutional base layers using new datasets can provide more reliable learning. Similar techniques are employed in ResNet50.

**Figure 2:**
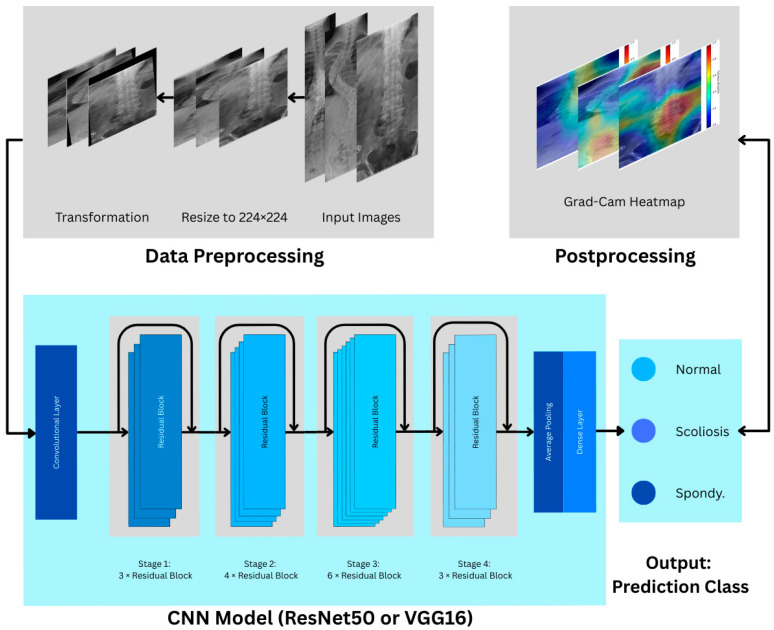
The visualization of the models’ classification pipeline.

Figure 2 displays the step-by-step process from the data preprocessing, where the input data is resized and transformed. Then, the preprocessed images are used as input for the CNN models. Finally, after obtaining the prediction class from the CNN model, we perform postprocessing analysis using Grad-CAM.

The hyperparameters used for both models are listed in Table 3. The models are trained using cross-entropy loss and adaptive moment estimation (Adam) [44]. Cross-entropy loss calculates the difference between expected class (output) and predicted class probabilities that are obtained from raw model outputs (logits). The logits are first converted into a probability distribution, where the sum of all probabilities equals one, using the softmax function. In Pytorch, this process is combined in the cross-entropy loss function, where cross-entropy loss is calculated by efficiently combining both softmax and Negative Log Likelihood (NLL) loss into a single step by directly taking the logits as input. The formula for cross-entropy loss is given in (1). For a batch containing N images, the cross-entropy loss is averaged as given in (2). In addition to the loss function, Adam is used as the optimizer of the models. The batch size chosen for training the models is 32. To get an optimal learning rate for each model, the models are trained using an initial learning rate of 0.0001. To improve the learning of the model, we applied a scheduler to reduce the learning rate when the validation loss plateaus after five epochs. The learning rate is reduced by a factor of 0.5 and is limited to a minimum learning rate of 1 × 10^−5^.

(1) $$L=-log(\frac{e^{x_{y}}}{\sum_{j}e^{x_{j}}}),$$

(2) $$Loss=-\frac{1}{N}\sum_{n=1}^{N}log(\frac{e^{x_{y}}}{\sum_{j}e^{x_{j}}})$$

In this paper, Grad-CAM is used to visualize the decision process within the deep learning models. The Grad-CAM is obtained by targeting the last convolution layer, where the feature maps generated by the deep learning models are interpreted. The heatmaps are generated after the model has been trained. To generate a heatmap for an image, the image is used as input to the model. The model then performs a forward pass to predict the specific spine abnormality class. Then, the gradients of the target class value with respect to the feature maps of the final convolutional layer are computed. These gradients are globally average-pooled to calculate the importance of weight in each feature channel. A weighted combination of the forward feature maps is then calculated and passed through a Rectified Linear Unit (ReLU) function to isolate the features that contribute to the class prediction. Finally, this coarse heatmap is upsampled to match the input image resolution and overlaid onto it. This heatmap overlay enables visual interpretation of the regions that contribute most to the class prediction, enabling medical professionals to validate the logic behind the predictions.

All experimental procedures in this study are conducted on a local workstation equipped with an RTX 2080 Ti with 11 GB of VRAM (NVIDIA Corporation, Santa Clara, CA, USA), an Intel i7-9700 processor (Intel Corporation, Santa Clara, CA, USA), and 64 GB of RAM. The deep learning architectures and Grad-CAM are implemented using Python (v3.10), Pytorch (v2.4.0), and TorchVision (v0.19.0).

### 3.2. Expert Analysis

To evaluate the validity of the classification results and predicted heatmaps, an evaluation was conducted through a structured survey involving a orthopedic specialist. Only incorrectly predicted cases by the fine-tuned models are included in this survey. The goal is to provide insight into models’ behavior, whether the failed cases are due to diagnostic complexity or the model’s inability to learn relevant features.

The survey is structured into three sections. The first section requires the expert to provide a clinical diagnosis for the images based on the original unprocessed images. The goal is to qualitatively evaluate the diagnostic difficulty of each case and whether a reliable diagnosis can be made only through visual inspection.

The second and third sections evaluated the Grad-CAM heatmaps of VGG16 and ResNet50, respectively. For each case, the expert assessed whether the model is able identify correct anatomical structures, and whether the attention is mainly on the structural features that are crucial for diagnosis. The feedback from the survey is analyzed to provide insight on the reliability of the proposed method for clinical diagnosis and interpretability.

## 4. Results and Discussion

This section provides the results of the classification task discussed in the methodology section. The result includes the classification performance of the transfer learning models, alongside the heatmap generated by implementing Grad-CAM on the trained models.

### 4.1. Overview of Models Classification Performance

The summary of the mean values of accuracy, sensitivity, precision, and F1-score across all folds is shown in Table 4. The classification performance metrics used are calculated using a macro average due to the class imbalance, as shown in Table 1. The result shows that the best-performing models are trained using fine-tuning. Fine-tuned VGG16 achieves a mean accuracy of 99.12% while fine-tuned ResNet50 achieves 98.22%. For both models, training from scratch outperformed the feature extraction models. The VGG16 trained from scratch presented a competitive mean accuracy of 93.79% compared to the ResNet50 scratch model, obtaining a significantly lower accuracy of 85.79%. Feature extraction VGG16 achieves slightly a higher mean accuracy of 77.23% compared to 74.26% on ResNet50 with feature extraction. VGG16 consistently achieves higher accuracy compared to ResNet50 on all training methods.

Both models gain a noticeable jump in performance when they are fine-tuned. ResNet50’s accuracy increased by around 24.0% while VGG16 gains 22.9%. Even though both methods utilized the same initial weights pretrained on the ImageNet dataset, freezing the convolutional layers produced significantly worse performance compared to updating the whole trainable parameters. This is likely contributed by the large domain gap between the ImageNet and our dataset. ImageNet consists of natural images compared to our medical dataset. However, the fine-tuning process is able to leverage the weights after all the weights are unfrozen. In addition to mean accuracy values, the fine-tuned models are more consistent, showing a variation accuracy of less than 2%. The variation is lower compared to other training methods, which range between 3.7% to 4.7%, indicating stable performance across all folds.

Furthermore, for training from scratch compared to fine-tuning, it is observed that the mean accuracy gain for ResNet50 is significantly higher compared to VGG16, with 12.4% and 5.33%, respectively. This may indicate that ResNet50, due to its deeper architecture, requires more data to learn the discriminative features for each class without any pretrained weights. Additionally, for ResNet50, it is observed that the variability of scratch models (±4.74%) is higher compared to feature extraction (±3.71%), even though the accuracy of the scratch model is higher. This shows lower generalizability of the scratch model across different splits. However, for VGG16, the scratch model shows an accuracy of 93.79%, 5.33% lower than the fine-tuned model. This is 8.0% higher compared to ResNet50, indicating that the model performs better for random weight initialization. This is contributed by the complexity of the model. VGG16 is an architecturally simpler model compared to ResNet50, with only 16 trainable layers compared to ResNet50 with 50 layers.

From Table 4, we can observe a trend among the feature extraction models across sensitivity, precision, and F1-score. It is observed that both feature extraction models obtain a sensitivity and F1-score of less than 70%. In contrast, the precision obtained a much higher value of around 87%. This is contributed by the class imbalance, where the number of scoliosis cases (*N* = 188) is much higher compared to normal (*N* = 71) and spondylolisthesis (*N* = 79). Because the weights are frozen and unable to learn the features of smaller classes, the models minimize the training loss by defaulting to the majority class. However, for other learning methods, this issue is not apparent, where the mean sensitivity and precision values are less than 2% apart.

While VGG16 obtains superior classification performance across all metrics, Table 5 highlights the computational trade-offs that contribute to these results. Both models are trained on an identical machine, batch size, and dataset size. The performance advantage of VGG16 comes with a higher cost in model complexity, with approximately five times the number of parameters and four times the FLOPs compared to ResNet50. This increased complexity and impacted computational efficiency. ResNet50 proves to be more efficient for training and inference, offering faster runtime per epoch (2.21 s vs. 2.65 s) and inference time (1.11 ms vs. 1.79 ms). However, for Grad-CAM heatmap generation, ResNet50 requires longer processing time for each image (16.37 ms per image) compared to VGG16 (13.73 ms per image). This difference may be related to architectural characteristics and gradient backpropagation complexity, although further analysis would be required to confirm this.

### 4.2. Performance of ResNet50

#### 4.2.1. Confusion Matrix

The confusion matrix result for all ResNet50 training strategies is displayed in Table 6. The confusion matrix is an aggregate of all five folds. Therefore, all prediction from 338 images in the dataset is included. The ResNet50 that is trained from scratch struggles to differentiate normal and scoliosis, where 19 normal spine images are predicted as scoliosis, and 15 scoliosis images are predicted as normal. The feature extraction model achieves the lowest accuracy of ResNet50. The model’s wrong predictions come mainly from the normal (*n* = 51) or spondylolisthesis (*n* = 33) images classified as scoliosis. The fine-tuned model shows the lowest wrong prediction, with the main errors being the normal spine identified as scoliosis (*n* = 3) and scoliosis spine identified as normal (*n* = 2). The prediction errors of feature extraction models highlight the model’s defaulting on the majority class due to class imbalance.

**Figure 3:**
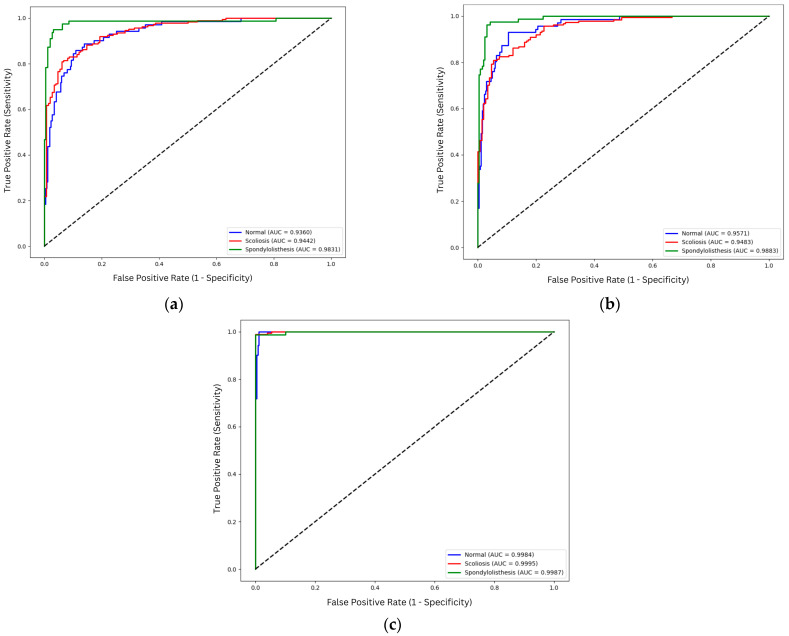
ROC curve of ResNet50 variations: (**a**) scratch; (**b**) feature extraction; (**c**) fine-tuned. The diagonal dotted line represents the performance of a random classifier (AUC = 0.5).

The receiver operating characteristic (ROC) curves shown in Figure 3 are based on the aggregated prediction of each class across all folds. Fine-tuned ResNet50 presents consistent classification with area under the curve (AUC) values exceeding 0.99 across all classes. For scratch and feature extraction, Spondylolisthesis achieves a higher AUC compared to Normal and scoliosis. This indicates better discriminative ability of the models for spondylolisthesis compared to other classes. On the other hand, the AUC values of normal and scoliosis are close to each other, ranging from 0.936 to 0.957.

However, comparing the performance of the ROC curves and the prediction results as shown in Table 4 and Table 6, significant discrepancies exist between the two results. Even though the AUC values are more than 0.93 for both training strategies, the mean accuracies for both feature extraction models are significantly lower compared to the scratch models. This discrepancy arises because the AUC evaluates the model’s ability to rank class probabilities across all thresholds, while the mean accuracy shown in Table 4 relies on the maximum probability prediction for each image provided by the softmax. Although feature extraction models produce relatively high AUC values, indicating the model’s ability to distinguish classes, the fixed decision threshold contributes to lower accuracy. Furthermore, the class imbalance amplifies this behavior, where the model tends to predict the majority class, reducing sensitivity despite good class discriminative ability. In short, high AUC does not necessarily translate to high classification accuracy.

#### 4.2.2. Grad-CAM Heatmap

**Figure 4:**
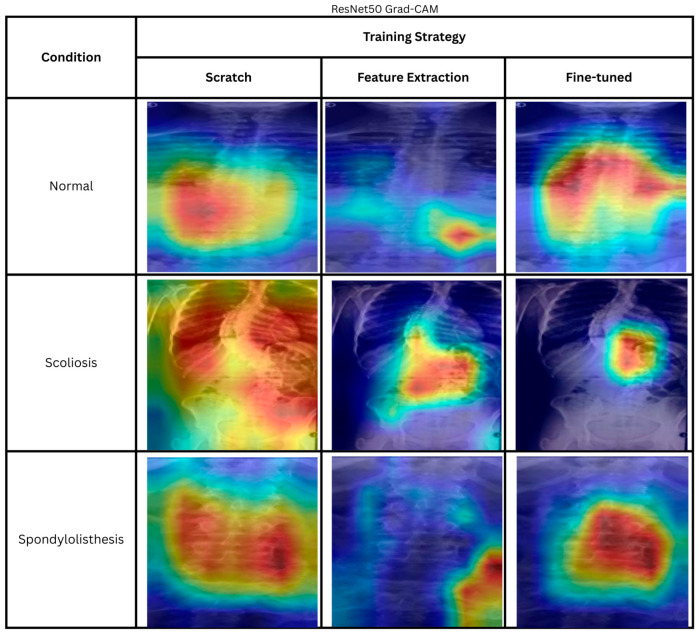
Heatmaps of correctly predicted cases using different ResNet50 training strategies. The color indicates the importance of local features for the model prediction, ranging from blue (low importance) to red (high importance).

The heatmaps of correctly predicted classes by ResNet50 trained from scratch, using the feature extraction method and fine-tuning method, are shown in Figure 4. The cases are randomly chosen. The scratch ResNet50 shows large regional activation in all three classes. The largest region activation is in the scoliosis class. The whole body is used to produce the prediction. As for the normal class, the model is focusing on the bottom left of the images or the side of the spine. On the other hand, the spondylolisthesis heatmap shows that the model is able to focus on the spine itself rather than the background.

The heatmaps from transfer learning feature extraction—normal and spondylolisthesis—show that the model is not able to focus on the correct regions important for the classification result. However, for scoliosis, the model is able to focus on the important feature of a scoliosis patient, the spine curvature. However, comparing the scratch and feature extraction method to the fine-tuned model, the fine-tuned model appears to focus more on the correct regions. In the normal image, the model focuses on the center of the spine; in the scoliosis image, it focuses on the area near the curve apex; and in the spondylolisthesis image, it concentrates on the spine segments. The results showcased that fine-tuned ResNet50 is generally better compared to the feature extraction and scratch method in focusing on diagnostically significant regions within the image.

The results shown by the feature extraction and training from scratch ResNet50 model highlight the trust issues in AI systems. Although the model correctly classified the images, this success was achieved accidentally, without actually focusing on the relevant areas. Therefore, XAI methods, such as Grad-CAM, are crucial for providing more transparent information on how an AI model makes its decisions, allowing for the selection of models that more accurately mimic human expert decision-making.

### 4.3. Performance of VGG16

#### 4.3.1. Confusion Matrix

The confusion matrix for VGG16 is displayed in Table 7. The fine-tuned model displays the fewest errors, with only an incorrect prediction for each class. The scratch has the second fewest errors with only eleven incorrect predictions. Most of the errors of scratch VGG16 are from normal spines predicted as scoliosis (*n* = 6). Lastly, the fine-tuned VGG16 achieves the fewest incorrect predictions, with only three images misclassified. Similar to the scratch ResNet50, the feature extraction model’s false predictions are mostly from normal (*n* = 45) and spondylolisthesis (*n* = 31) images that are predicted as scoliosis. This is likely contributed to by class imbalance, where the loss optimization function defaulted on the majority class, inflating the number of errors.

**Figure 5:**
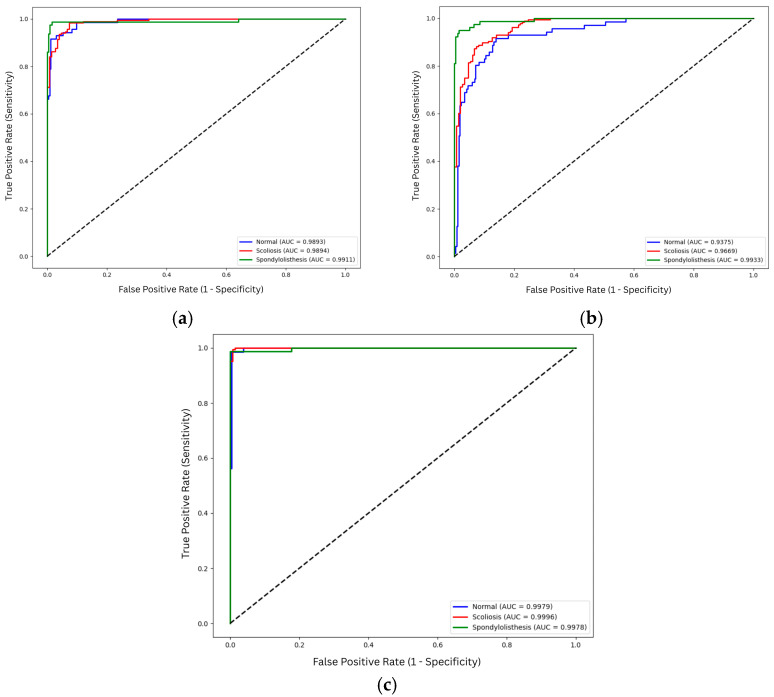
ROC curve of VGG16 variations: (**a**) scratch; (**b**) feature extraction; (**c**) fine-tuned. The diagonal dotted line represents the performance of a random classifier (AUC = 0.5).

The ROC curves for VGG16 as shown in Figure 5, further highlight the impact of training strategies and architectural differences. Fine-tuned configurations produce the highest AUC on all classes for VGG16. Scoliosis obtains an almost perfect AUC of 0.9996, while normal and spondylolisthesis also display high AUCs of 0.9979 and 0.9978, respectively. Similar to ResNet50, the feature extraction approach for VGG16 achieves the lowest AUC for scoliosis and normal classes, while the model is able to discern spondylolisthesis from other classes properly with an AUC value of 0.9933.

In contrast to ResNet50, the VGG16 model trained from scratch displays higher discriminative abilities across all the training strategies. The spondylolisthesis achieves the highest AUC value for scratch VGG16 with 0.9911, followed by 0.9894 for scoliosis images, and 0.9893 for normal images. This stability may be influenced by the VGG16 simpler architecture that is able to learn from a restricted dataset as compared to the deeper architecture of ResNet50.

#### 4.3.2. Grad-CAM Heatmaps

**Figure 6:**
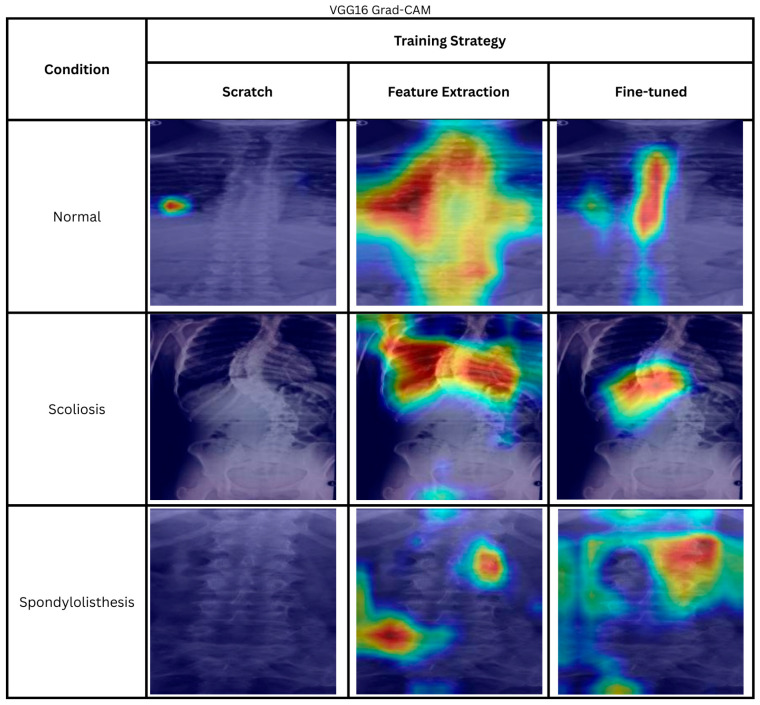
Heatmaps of correctly predicted using different VGG16 training strategies. The color indicates the importance of local features for the model prediction, ranging from blue (low importance) to red (high importance).

The heatmaps shown in Figure 6 include the correct predictions of VGG16 models. The Grad-CAM heatmap shows that the model produces minimal activation when trained from scratch. On the other hand, the VGG16 feature extraction heatmaps target anatomically relevant regions for normal and scoliosis. Similarly, fine-tuned VGG16 displays a heatmap pattern that focuses on the spine itself. However, if we compare both training strategies for normal and scoliosis heatmaps, we can see that the fine-tuned model focuses on a smaller region compared to feature extraction. For the normal spine image, the heatmap for the fine-tuned model tightly focuses on the spinal column, while the feature extraction model focuses not only on the spinal column but also on the neighboring organs. Likewise, the scoliosis images of feature extraction do not cover only the spine but also neighboring organs. In contrast, the fine-tuned model focuses on the spinal column’s apex, a defining feature of scoliosis. However, for all VGG16 training strategies, the model struggles to focus on the spine for the spondylolisthesis image.

From the observation, even though the scratch VGG16 achieves a high accuracy of 93.79%, the Grad-CAM heatmap is not interpretable, thus limiting the explainability of the model. On the other hand, the fine-tuned VGG16 produces relevant heatmaps for normal and scoliosis images that focus on the relevant features, which enable the user to observe the relevance of the prediction. Once again, the observations made here show the importance of explainability to promote trust in the AI decision. 

Cho et al. implemented similar training techniques as our fine-tuned model [45]. The model was pretrained using the ImageNet dataset and fine-tuned using their own dataset of fundus photographs of patients with normal or myopic tilted disk. Through heatmaps, the authors proved that the model is able to correctly interpret the images by locating important regions for tilted optic disk classification, which are the retinal sections. Correlations between important regions that the model focused on may assist the clinician in determining the relevance of the classification result. Heatmap visualization is also being used by Ausawalaithong et al. to visualize the decision process done by three deep learning models for lung cancer prediction [46]. The authors found that one of the models is able to locate the area of the lung with cancer better than the other models.

### 4.4. Expert Clinical Interpretation

**Figure 7:**
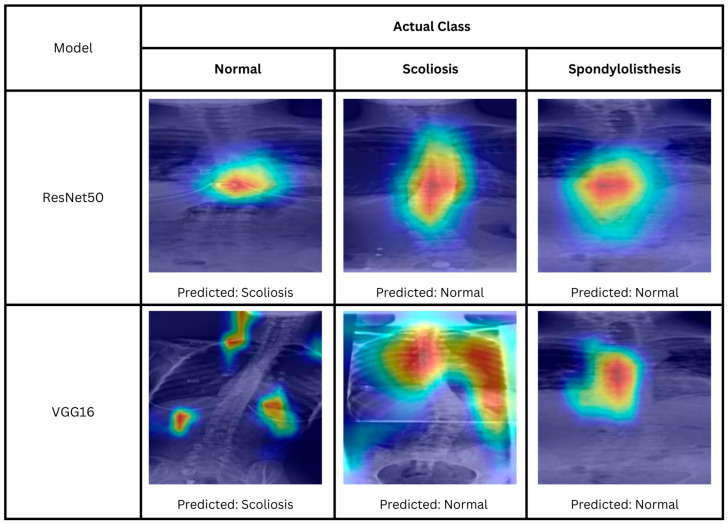
Heatmaps of correctly predicted normal, scoliosis, and spondylolisthesis using fine-tuned ResNet50 and fine-tuned VGG16. The color indicates the importance of local features for the model prediction, ranging from blue (low importance) to red (high importance).

As shown in Table 4, fine-tuned models achieved the best performance compared to other configurations. In Table 7, there are three incorrectly predicted cases by VGG16, while in Table 6, there are six incorrectly predicted cases by the fine-tuned Resnet50. All cases are analyzed by the expert using the survey discussed in Section 3.2. Figure 7 displays the comparison of a representative sample for each actual prediction class for each model. As VGG16 only has three incorrect predictions, all the errors are displayed. For both models, it is observed that they struggle to identify a similar case of spondylolisthesis. Both models predicted the image as a normal spine. The expert evaluated the case and concluded it as a mild scoliosis. According to the expert, the listhesis is best viewed on the lateral image rather than frontal view of the dataset. Comparing both heatmaps, although they focus mainly on the spine itself, the models are unable to identify the location of the listhesis, resulting in incorrect predictions.

For the normal case, VGG16 predicted a normal spine as scoliosis. The model’s heatmaps shows that it focuses on the non-spine region of the image explaining why the case is wrongly classified. For ResNet50, the model predicted an image labeled normal as scoliosis. Interestingly evaluation by the expert, the case is found to exhibit a trait of mild scoliosis; however, he noted that further measurement is required, as it is a boundary case. In the heatmap of ResNet50, the model identifies a trait of a scoliosis spine, that is, the apex of the spine curvature. This indicates that even though the image is originally labeled as a normal spine, the model is identifying the correct feature of the class prediction.

The last class is scoliosis. For VGG16, we can see that the model focuses not only on the spine but also on the lungs and ribs. The model is unable to locate the clinically relevant region for diagnosis and predicted it as normal. However, for ResNet50, the model does not provide a correct prediction. Nonetheless, the model was able to correctly identify the spine. The expert diagnoses the case and concludes as moderate scoliosis where the curve is not prominent.

Even though VGG16 achieved better accuracy, the model heatmaps were assessed by the expert as having less clinically relevant localization compared to ResNet50. Although some errors are due to the difficulty of diagnosis, most of the incorrect predictions (two out of three) by VGG16 happen due to the model’s failure to locate features relevant for classification. In contrast, even though ResNet50 achieved slightly lower accuracy, the heatmaps of the incorrect predictions were more consistent with expert interpretation, suggesting more reliable feature localization.

### 4.5. Comparison with Past Works

Table 8 summarizes previous studies on scoliosis and spondylolisthesis classification using deep learning models. Several studies report high classification performance, with accuracy ranging from 92% to 99% [30,31]. However, these approaches do not incorporate XAI techniques, limiting the interpretability of the model predictions. Similarly, a study used a fine-tuned VLM for multi-stage classification of spine scoliosis [32]. However, the model only outputs the model’s class prediction without a textual description or additional interpretability methods.

Some studies have attempted to improve interpretability by integrating XAI methods such as Grad-CAM. For example, Takahashi et al. applied Grad-CAM with an ensemble model for binary classification, but reported inconsistent activation patterns and relatively lower performance, with 70.4% accuracy [34]. Chen et al. achieved 92% accuracy for multiclass classification with Grad-CAM interpretation assisted by medical experts; however, the evaluation was limited to a single model and training configuration [33]. Aside from Grad-CAM, partition-based explanation methods have been proposed to identify discriminative local features for binary and multiclass classification [35], although the validity of the generated visualizations from the partition explainer was not validated through expert assessment. Despite these efforts, systematic evaluation of model training strategies together with explainability analysis remains limited in the current literature.

In contrast, our proposed methods present competitive accuracy of 74.26% to 99.12% while providing comprehensive training strategies. Whereas most prior studies primarily relied on transfer learning through fine-tuning, this study evaluates three training strategies in both classification performance and explainability using Grad-CAM. Furthermore, Grad-CAM visualizations are analyzed for both correct and incorrect predictions, with the assistance of a medical expert’s interpretation for incorrect predictions. The analysis provides additional insight into the models’ decision behavior, providing deeper insight into model decision behavior and supporting more clinically interpretable predictions.

The proposed methods obtain a competitive performance, comparable with previous studies on scoliosis and spondylolisthesis classification. Even though some past studies display high classification accuracy, the presented methodology only includes a single training strategy without extensive evaluation on the explainability of the results. Conversely, this study presents not only the performance evaluation on multiple training strategies on the selected models but also analyzes them under a consistent experimental environment.

The factors that contribute to the advantage of this study include the implementation of five-fold cross-validation. The training method enables a more rigorous estimation of the model performance, particularly on a small dataset (*N* = 338), by reducing sample bias and improving generalization ability. In addition, the models’ training configurations, including the adaptive learning rate scheduling (ReduceLROnPlateau), early stopping, and optimized hyperparameters, assist in enabling stable convergence and reducing overfitting, which is crucial for small dataset classification on medical imaging data. Next, the highest-performing models (Accuracy > 98%) utilize the fine-tuning of pretrained data on large natural images, while adapting to domain-specific spinal features, resulting in competitive classification compared to training from scratch and feature extraction.

While most past studies focus mainly on classification performance, this study provides additional insight into the explainability analysis of the established CNN architectures, which are VGG16 and ResNet50. The Grad-CAM heatmap is presented for both correct and incorrect predictions, with an analysis of the regions of activation. Additionally, medical experts’ interpretations are provided on incorrectly predicted images, providing additional insight into the features that the model would incline to produce in incorrect predictions.

### 4.6. Limitations and Future Work

The primary limitation of this study is the relatively small sample size (*N* = 338) compared to large-scale natural image datasets. While the deep learning models commonly require a large set of data to generalize effectively, we mitigated this limitation by employing transfer learning (pretrained on ImageNet weights) and rigorous stratified 5-fold cross-validation. Although our high accuracy and ROC scores suggest robust learning, the risk of overfitting cannot be entirely ruled out without testing on a larger dataset. Future work will focus on expanding the dataset to improve the class balance and include a wider variety of spinal morphologies to validate the model’s stability further.

Secondly, the dataset is from a single institution, which introduces potential selection bias regarding the patient demographics, X-ray machine used, and image acquisition protocols. Consequently, the model’s generalizability to external datasets remains to be verified. To solve this, future research should involve a multi-center study to test the model’s performance on X-ray images from multiple clinical sources, ensuring that the features learned (from the Grad-CAM) are universal and not specific to a dataset.

Finally, while the proposed models successfully classify spinal conditions, they do not currently provide quantitative measurements, such as Cobb angle (scoliosis diagnosis), or the degree of vertebral slippage (spondylolisthesis). In a clinical setting, these metrics are crucial for surgical planning. Therefore, this study serves as a foundational screening tool that can assist in the diagnosis process.

## 5. Conclusions

This study aims to assess the relevance of Grad-CAM heatmaps generated by transfer learning methods for VGG16 and ResNet50 in the analysis of spine abnormalities. The findings support the importance of XAI in developing trustworthy AI-based medical analysis systems. An analysis based solely on traditional performance metrics such as accuracy, sensitivity, precision, and F1-score can be misleading. For instance, the accuracy of VGG16 trained from scratch, where the weights of all the convolutional layers are frozen, is 93.79%. However, the heatmaps generated from training from scratch methods show that the regions influencing the classification results do not align with those necessary for the diagnostic task performed by medical professionals. The heatmaps generated from fine-tuning for both models display better identification of clinically important regions. Comparing ResNet50 with VGG16 reveals that the area examined by ResNet50 closely matches the area focused on by medical experts when making assessments of the X-ray images.

Therefore, the usage of XAI in this work suggests that, for AI-based spine abnormality analysis, the fine-tuned ResNet50 is the most suitable model. In transfer learning, fine-tuning a model for a new task is necessary to ensure it adapts to the distinctive features of the new dataset. Additionally, incorporating XAI in critical areas such as healthcare is essential to ensure that the developed systems are transparent and trustworthy.

## Figures and Tables

**Table 1:** Dataset class information.

Spine Diagnosis	Total	Female	Male
Normal	71	40	31
Scoliosis	188	151	37
Spondylolisthesis	79	49	30

**Table 2:** Training strategies summary.

Training Strategy	Pretrained Weights	Frozen Layers	Trainable Layers
Scratch	No	None	All
Feature Extraction	Yes (ImageNet)	All except dense layers	Dense Layers Only
Fine-tuning	Yes (ImageNet)	None	All

**Table 3:** The hyperparameters for ResNet50 and VGG16.

Hyperparameters	Configurations
Optimizer	Adam
Learning Rate	1 × 10^−4^
Batch Size	32
Num. Epochs	50
Loss Function	Cross-Entropy Loss
Early Stopping	10
Scheduler	Reduce LR on Plateau

**Table 4:** Classification results summary.

Model	Training Method	Accuracy (%)	Sensitivity (%)	Precision (%)	F1-Score (%)
	Scratch	85.79 (±4.74)	83.96 (±4.58)	85.26 (±5.66)	84.47 (±5.02)
ResNet50	Feature Extraction	74.26 (±3.71)	60.97 (±5.59)	87.10 (±4.04)	64.11 (±6.93)
	Fine-tuned	98.22 (±1.74)	97.84 (±1.89)	98.14 (±2.08)	97.96 (±1.94)
	Scratch	93.79 (±3.44)	93.33 (±3.63)	93.36 (±3.25)	93.22 (±3.47)
VGG16	Feature Extraction	77.23 (±3.86)	65.27 (±5.95)	87.13 (±4.71)	69.23 (±6.99)
	Fine-tuned	99.12 (±1.18)	98.96 (±1.67)	98.94 (±1.33)	98.93 (±1.47)

**Table 5:** Computational complexity summary.

Model	Number of Parameters	FLOPs	Runtime (Per Epoch)	Inference Time (Per Image)	Grad-CAM (Per Image)
VGG16	138.36 M	15.5 G	2.65 s	1.79 ms	13.73 ms
ResNet50	25.56 M	3.8 G	2.21 s	1.11 ms	16.37 ms

**Table 6:** Confusion matrix summary of ResNet50 using training from scratch, feature extraction, and fine-tuning.

Training Strategy	Class		Predicted		
			Normal	Scoliosis	Spondylolisthesis
Scratch	Actual	Normal	51	19	1
		Scoliosis	15	167	6
		Spondylolisthesis	0	7	72
Feature Extraction	Actual	Normal	19	51	1
		Scoliosis	0	187	1
		Spondylolisthesis	1	33	45
Fine-Tuning	Actual	Normal	68	3	0
		Scoliosis	2	186	0
		Spondylolisthesis	1	0	78

**Table 7:** Confusion matrix summary of VGG16 using training from scratch, feature extraction, and fine-tuning.

Training Strategy	Class		Predicted		
			Normal	Scoliosis	Spondylolisthesis
Scratch	Actual	Normal	64	6	1
		Scoliosis	1	185	2
		Spondylolisthesis	1	0	78
Feature Extraction	Actual	Normal	26	45	0
		Scoliosis	0	188	0
		Spondylolisthesis	4	31	44
Fine-Tuning	Actual	Normal	70	1	0
		Scoliosis	0	187	1
		Spondylolisthesis	1	0	78

**Table 8:** Comparison with past work.

Ref.	Spine Pathology	XAI Methods	Classification Type	Training Strategy	Model	Accuracy
[30]	Scoliosis, Spondylolisthesis	None	Multiclass	Transfer Learning (FT)	AlexNet, GoogleNet, ResNet, EfficientNet	92.65–99.01%
[31]	Spondylolisthesis	None	Binary	Transfer Learning (FT)	InceptionV3, VGG16	96–98%
[32]	Scoliosis	None	Multi-stage	Transfer Learning (FT)	ViT, PubmedBERT	53–80%
[33]	Spondylolisthesis	Grad-CAM	Multiclass	Transfer Learning (FT)	EfficientNetV2	92%
[34]	Scoliosis	Grad-CAM	Binary (Ensemble)	Transfer Learning (FT)	ViT, SwinT, ConvNextV2, InceptionV3, DenseNet121, ResNet50	70.4%
[35]	Scoliosis, Spondylolisthesis	Partition Explainer	Binary and Multiclass	Transfer Learning (FT)	Xception	99.14%
Ours	Scoliosis, Spondylolisthesis	Grad-CAM	Multiclass	Scratch, Transfer Learning (FE and FT)	VGG16, ResNet50	74.26–99.12%

(Note: FE = Feature Extraction; FT = Fine-Tuning).

## Data Availability

This paper uses data collected by researchers of Jordan University of Science and Technology from the King Abdullah University Hospital, Jordan. The data is reported in Fraiwan et al. [47]. Data are publicly available for researchers: https://www.kaggle.com/datasets/yasserhessein/the-vertebrae-xray-images (accessed on 1 October 2024).
